# Heart Rate Dynamics During Acute Recovery From Maximal Aerobic Exercise in Young Adults

**DOI:** 10.3389/fphys.2021.627320

**Published:** 2021-02-05

**Authors:** Nathaniel T. Berry, Emily Bechke, Lenka H. Shriver, Susan D. Calkins, Susan P. Keane, Lilly Shanahan, Laurie Wideman

**Affiliations:** ^1^Department of Kinesiology, The University of North Carolina at Greensboro, Greensboro, NC, United States; ^2^Department of Nutrition, The University of North Carolina at Greensboro, Greensboro, NC, United States; ^3^Department of Human Development and Family Studies, The University of North Carolina at Greensboro, Greensboro, NC, United States; ^4^Department of Psychology, The University of North Carolina at Greensboro, Greensboro, NC, United States; ^5^Department of Psychology, University of Zurich, Zurich, Switzerland

**Keywords:** heart rate variability, non-linear dynamics, recovery heart rate, recovery heart rate variability, cardiac dynamics, emerging adults

## Abstract

**Introduction:**

Resting heart rate (HR_rest_), heart rate variability (HRV), and HR recovery (HRR) from exercise provide valuable information about cardiac autonomic control. RR-intervals during acute recovery from exercise (RR_rec_) are commonly excluded from HRV analyses due to issues of non-stationarity. However, the variability and complexity within these trends may provide valuable information about changes in HR dynamics.

**Purpose:**

Assess the complexity of RR_rec_ and determine what physiologic and demographic information are associated with differences in these indices in young adults.

**Methods:**

RR-intervals were collected throughout maximal treadmill exercise and recovery in young adults (*n* = 92). The first 5 min of RR_rec_ were (1) analyzed with previously reported methods that use 3-interval lengths for comparison and (2) detrended using both differencing_(diff)_ and polynomial regression_(res)_. The standard deviation of the normal interval (SDNN), root mean square of successive differences (rMSSD), root mean square (RMS) of the residual of regression, and sample entropy (SampEn) were calculated. Repeated measures analysis of covariance (ANCOVA) tested for differences in these indices for each of the methodological approaches, controlling for race, body fat, peak oxygen uptake (VO_2p__eak_), and resting HR (HR_rest_). Statistical significance was set at *p* < 0.05.

**Results:**

VO_2p__eak_ and HR_rest_ were significantly correlated with traditional measures of HRR and the variability surrounding RR_rec_. SampEn_diff_ and SampEn_res_ were correlated with VO_2p__eak_ but not HR_rest_ or HRR. The *residual-*method provided a significantly (*p* = 0.04) lower mean standard error (MSE) (0.064 ± 0.042) compared to the *differencing-*method (0.100 ± 0.033).

**Conclusions:**

Complexity analysis of RR_rec_ provides unique information about cardiac autonomic regulation immediately following the cessation of exercise when compared to traditional measures of HRR and both HRrest and VO2peak influence these results.

## Introduction

Historically, the response of heart rate (HR) and heart rate recovery (HRR) to- or following- a perturbation, has been commonly utilized within research and clinical settings as a non-invasive physiological measure of cardiovascular regulation. Several studies have shown that impairment in autonomic function—represented by delayed HRR—indicates adverse cardiovascular outcomes in otherwise healthy individuals (Qiu et al., 2017; Lachman et al., 2018). While changes in HR and traditional indices of HRR can provide important information about differences in cardiac autonomic control, heart rate variability (HRV) has been shown to offer a more sensitive measure of cardiac autonomic regulation at rest (Task-Force, 1996) and during exercise (Karapetian et al., 2008) compared to a mean HR value. As measures of HR, HRR, and HRV have clear associations with cardiovascular health, a better understanding of RR intervals following various exercises and exercise intensities during the acute recovery phase (RR_rec_) is needed within the literature. Doing so, provides implications for clinical, research, and performance-related exercise testing and monitoring.

Michael et al. (2017) highlight and discuss the usefulness of assessing HRV at the onset, during, and immediately following the cessation of exercise to provide insight into autonomic stress reactivity. They, Michael et al. (2017), suggest that upon the onset of exercise, inputs from higher-order brain centers feed into the medullary control of cardiovascular function to reset the arterial baroreflex. This shift in the operating point for the arterial baroreflex requires a physiological shift in cardiac output that is met primarily by a withdrawal in parasympathetic input to increase HR. HRV provides context to changes in cardiac regulation beyond the assessment of mean HR values but these methods are limited to stationary time-series. The statistical estimation of a time-series (whether that statistic is assessing the variability or the complexity of the time-series) is dependent upon the underlying process producing the time-series and because each element of a non-stationary time-series can have a different entropy, these statistical calculations become consistently biased (Berry et al., 2020). Prior research has examined the relations between changes in HRV and the non-stationary (data with significant slopes in the HR response) trends in exercise recovery with conflicting HRV results (Arai et al., 1989; Bernardi et al., 1990; Perini et al., 1990; Breuer et al., 1993; Nakamura et al., 1993; Oida et al., 1997; Perini and Veicsteinas, 2003).

While a few studies have investigated methodological approaches for HRV indices during and/or after exercise, these studies have produced conflicting results (Arai et al., 1989; Bernardi et al., 1990; Perini et al., 1990; Breuer et al., 1993; Nakamura et al., 1993; Oida et al., 1997; Perini and Veicsteinas, 2003). As it pertains to exercise recovery, these methods are less commonly reported in the literature than traditional measures of HR and HRR. A recent review by Peçanha et al. (2017) examined the strengths and weaknesses of a variety of methods that have previously been utilized to assess post-exercise autonomic recovery via HRR and HRV indices. As discussed in this review (Peçanha et al., 2017), Goldberger et al. (2006) first introduced the idea of separating-out the 5 min period following the cessation of exercise into twenty 15s, ten 30s, and five 60s segments, which could be assessed by different variability indices to evaluate changes in cardiac autonomic reactivation. The piecewise analysis of these first 5 min following the cessation of exercise utilized HRV indices such as the standard deviation of normal RR-intervals (SDNN), the root mean square of successive RR differences (rMSSD), and the root mean square (RMS) (Ganio et al., 2009) index to assess cardiac autonomic reactivation. The authors concluded that both rMSSD and RMS of 30 s segments adequately reflected the autonomic changes occurring during the 5 min period of recovery (compared to 15 and 60 s segment lengths) and eventually expanded on these findings by evaluating these methods following a submaximal exercise bout (Ng et al., 2009). These studies show that indices of HRR and HRV immediately following the cessation of exercise reflect changes in cardiac autonomic modulation—as was also discussed in Michael et al. (2017).

However, measures of complexity—approximate entropy (ApEn) and sample entropy (SampEn), were not assessed in previous studies, but reflect important changes in heart rate dynamics that are not detected by traditional linear or frequency based measures of HRV (Goldberger Ary et al., 2000). Briefly, complexity measures stem from evaluation of non-linear dynamics, which assess the structure of a time series and reflects the system’s ability to adapt to internal or external perturbations. For instance, entropy indices quantify the degree of regularity or the appearance of repetitive patters within a time segment vs. traditional linear measures that examine the magnitude of variability. Thus, a low amount of entropy corresponds to a high amount of regularity within a signal (i.e., low adaptability of the system) vs. a higher degree of complexity that reflects a less-ordered signal (i.e., increased adaptability of the system)(West and Goldberger, 1987; Manor et al., 2010). A recent study by Berry et al. (2020), expanded on previous research by examining the influence of non-stationarity on indices of variability and complexity as well as various methods of detrending these data. These findings clearly show the biasing effects of non-stationarity on measures of variability and complexity as well as the effects of various methods of detrending on these indices.

To further elucidate the changes in heart rate dynamics immediately post-exercise, this study assesses the complexity during the entire 5 min recovery period immediately post-exercise using both a previously reported method of assessing HRV during the recovery from exercise (Goldberger et al., 2006) and the methods outlined in Berry et al. (2020). In addition, we examine the relations between various physiological measures with indices of HRV from each of the respective methods.

## Materials and Methods

This analysis was performed on data that had been collected as part of the larger longitudinal RIGHT Track (RT) Health study. The overarching aim of RT Health is to examine the influence of early childhood self-regulation on the development of cardiovascular risk in adolescence and young adults. This study was approved by the Institutional Review Board at the University of North Carolina at Greensboro (IRB #11-0360). Prior to participating, all subjects provided written and informed consent.

The methods outlined below are specific to the HRV assessments and analyses; methods on the entire study have been published elsewhere (Wideman et al., 2016). Participants arrived at the laboratory between the hours of 0900 and 1400 at least 2 h post-prandial and having abstained from moderate to vigorous physical activity for 24 h, caffeine for 12 h, alcohol for 48 h. Body composition was assessed via air displacement plethysmography using COSMED’s BODPOD^®^ system. An incremental test to exhaustion was performed on a GE 2100 treadmill to assess peak oxygen uptake (VO_2p__eak_) (Parvo Medics TrueOne 2400). Pre-exercise, during exercise, and post-exercise RR intervals were recorded using a Polar V800 watch and downloaded following the completion of the visit. During the exercise test, participants self-selected their running speed while grade was increased by 3% every 2 min. Participants were asked to choose a speed that they felt comfortable maintaining for approximately 15 min so that the incremental increases in grade would result in maximal volitional fatigue within 8–12 min.

**FIGURE 1:**
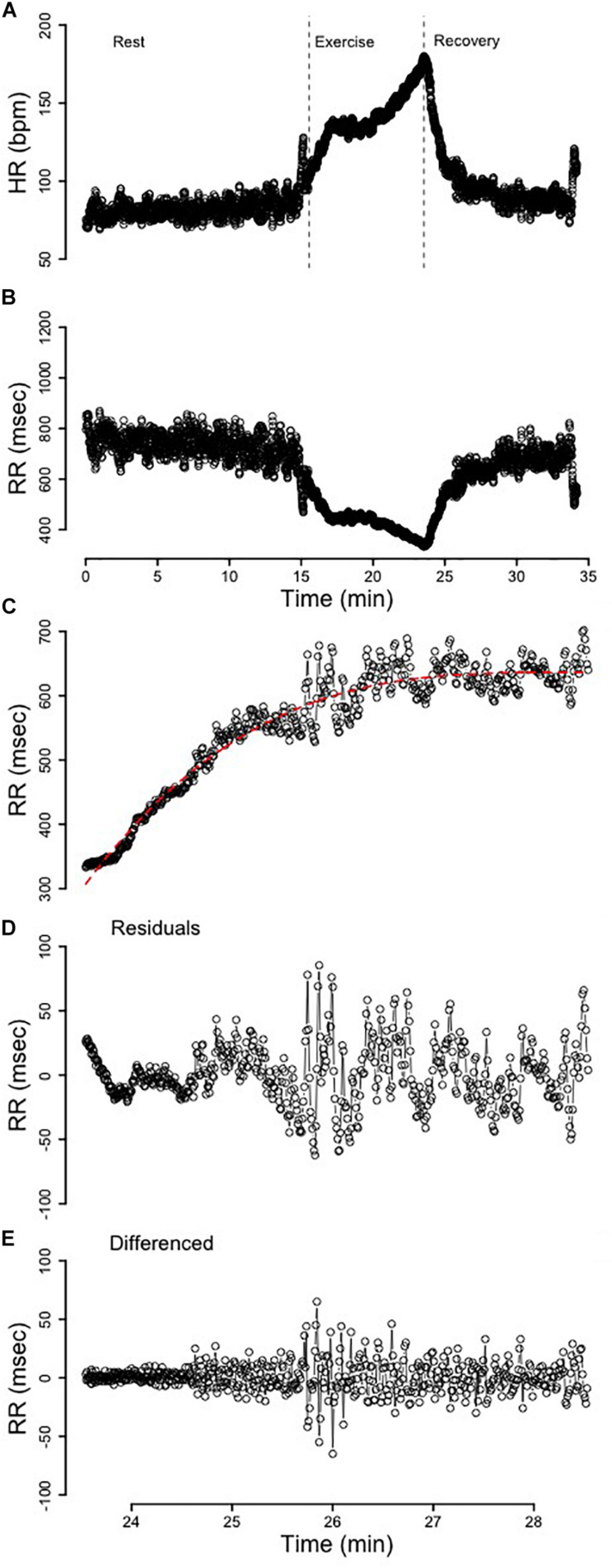
**(A)** The heart rate (HR) profile from rest, exercise, and recovery. The dotted vertical lines segment the immediate 5 min of recovery. **(B)** The RR-interval profile from rest, exercise, and recovery. **(C)** The 5 min of recovery RR-interval data fit with a third-order polynomial. **(D)** The residuals from the third-order polynomial in C that were used for analysis. **(E)** The 5 min of recovery RR-interval data detrended via differencing.

All mathematical and statistical procedures were performed using R 3.5.0. Artifact correction using linear interpolation of preceding- and proceeding- heart periods was completed prior to any additional analyses being performed using the open source “RHRV” package (Rodríguez-Liñares et al., 2008). Analysis of variance was performed using the “car” package (Fox and Weisberg, 2019) while multivariate adaptive regression splines were performed using the “earth” package (Milborrow, 2017). A example of an entire HR (bpm) and RR-interval (msec) profile is provided in Figures 1A,B, respectfully.

### Replication of Methods Outlined by Goldberger et al. (2006)

**FIGURE 2:**
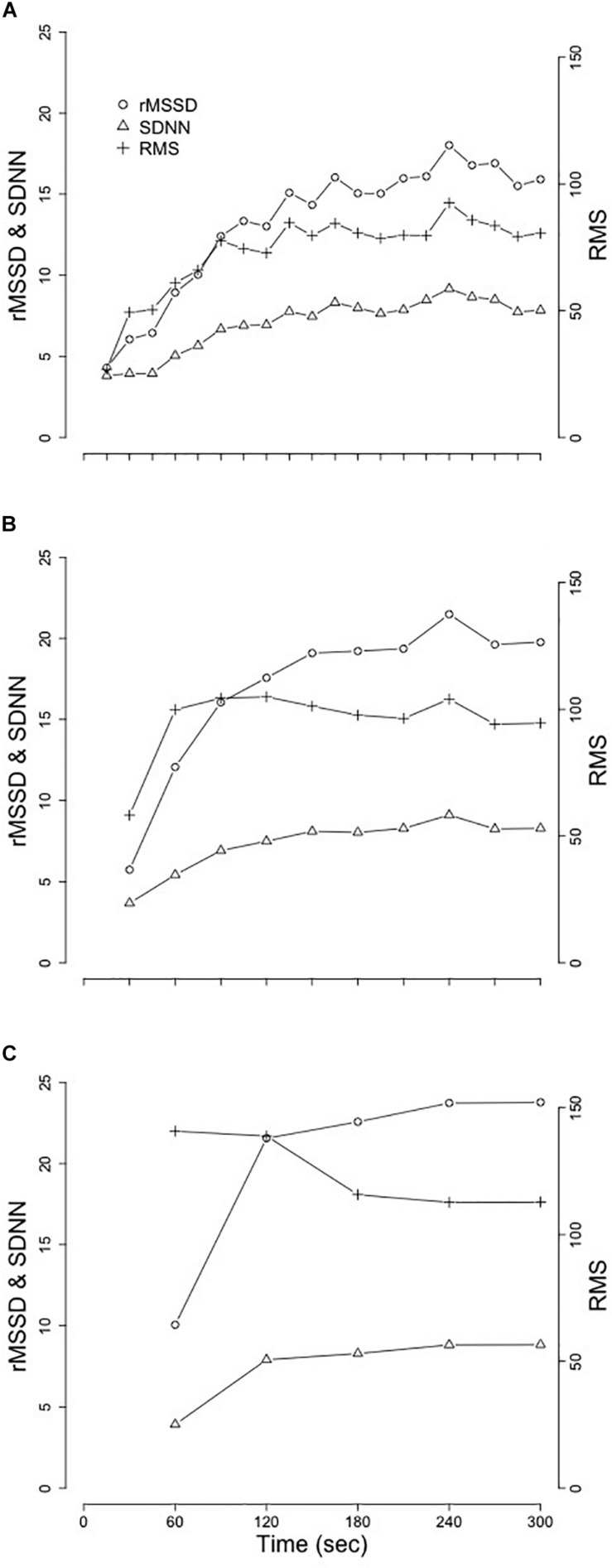
Mean changes of SDNN, rMSSD, and RMS from the replication of Goldberger et al. (2006). **(A)** 15 s segment. **(B)** 30 s segments. **(C)** 60 s segments. Standard deviation of the normal RR-interval (SDNN); root mean square of successive differences (rMSSD); root mean square (RMS).

The first 5 min of RR-interval data following the cessation of exercise were identified (Figures 1B,C). These segments were broken apart into twenty 15s, ten 30s, and five 60s segments that spanned the entire 5-min of recovery (Figure 2). Following linear regression analysis on the RR-intervals for each of the three different segment lengths, the residuals were computed and corresponding SDNN, rMSSD, and RMS indices were calculated.

### Differencing and Polynomial Detrending of RR_rec_

Two separate detrending approaches were performed and comparisons between these two methods were evaluated. These methods of detrending non-stationary time-series are provided in Berry et al. (2020). *Difference(_diff_) Calculations*. Differencing (Figure 1E) was used to detrend the time-series and the resultant stationary time-series was subsequently analyzed to compare differences in variability and complexity. *Residual(_res_) Calculations*. The optimal model was determined by purposefully overfitting the initial 5 min of RR_rec_ data for all subjects. After modeling the initial 5 min of RR_rec_ from 1 to 10 orders for each individual, a third-order polynomial was determined to be the optimal regression line (Figure 1C), since it was flexible enough to account for individualities in this response and reduced the autocorrelation among the residuals without significantly overfitting the data. The residuals from these *(individualized)* lines were plotted as a secondary time-series (Figure 1D) for analysis.

### Variability and Complexity of the Detrended RR_rec_ Data

The aforementioned transformations of the data were completed with the purpose of creating a time-series with a stationary mean to analyze the variability and complexity surrounding RR_rec_; allowing us the ability to characterize the patterns of variability around the non-stationary decline in HR (rise in RR-intervals). *Variability Measures.* The equivalent of common time-domain measures of HRV, including SDNN of normal RR intervals and rMSSD, were used to assess the variability of the detrended-RR_rec_. *Complexity Measures.* ApEn and SampEn were used to assess the complexity of the residuals surrounding the non-linear trend in RR_rec_; a higher number indicates a greater degree of complexity, whereas smaller values characterize more regular signals. While SampEn is more robust against changes in data-length compared to ApEn (Yentes et al., 2013), both indices were included in this analysis for continuity with previously published data.

### Statistical Methods

With respect to our replication of Goldberger et al. (2006), repeated measures analysis of variance (ANCOVA) were used to assess changes in SDNN, rMSSD, and RMS across time between sex after controlling for race, body fat (BF), VO_2p__eak_, and HR_rest_. Separate univariate ANCOVAs were used to compare differences in SDNN_diff_, rMSSD_diff_, SampEn_diff_, SDNN_res_, rMSSD_res_, and SampEn_res_ between sex and race after controlling for changes in BF, VO_2p__eak_, and HR_rest_. After these statistical comparisons were performed, exploratory statistical learning-methods were used to better understand some of the relations among these indices with various physiologic/demographic data.

Multivariate adaptive regression splines (ARS) produce continuous derivatives and are not only more powerful but also more flexible in determining the interaction among variables (Friedman, 1991). The original dataset was split, via random sampling, into a *training-dataset* (*n* = 46) and a *testing-dataset* (*n* = *46)*. Subsequently, the selected model was tested on the remaining data (the *testing-dataset*) to determine the validity of the models produced with the training-dataset. Physiological measures considered for each model included: height (Ht), weight (Wt), body fat (BF), body mass index (BMI), maximal oxygen uptake (VO_2p__eak_), resting HR (HR_rest_), maximal HR (HR_max_), HR at 1 min (HR_one_), and 5 min (HR_five_) following the cessation of exercise, recovery (Δ bpm) at 1 min (HRR_one_) and at 5 min (HRR_five_). These measures were scaled and centered prior to being used within the ARS analysis.

## Results

Data from *N* = 92 young adults (male = 46, female = 46) were included in this study. Subject demographics, separated by sex, are provided in Table 1.

**TABLE 1:** Subject demographics.

	**Males**		**Females**		**Combined**	
	***n* = 46**		***n* = 46**		***N* = 92**	
Age (years)	18.5	(± 0.5)	18.5	(± 0.6)	18.5	(± 0.6)
Height (cm)	178.0	(± 8.1)	164.7	(± 7.1)	171.4	(± 10.1)
Weight (kg)	74.8	(± 15.7)	69.3	(± 16.1)	72.1	(± 16.4)
BF (%)	16.8	(± 9.0)	30.8	(± 9.8)	23.8	(± 11.7)
BMI (kg/m^2^)	23.6	(± 4.4)	25.6	(± 5.8)	24.6	(± 5.2)
VO_2_ (ml/kg/min)	52.5	(± 10.0)	36.2	(± 9.4)	44.4	(± 12.7)
HR_rest_ (bpm)	55	(± 12)	62	(± 10)	59	(± 12)
HR_max_ (bpm)	199	(± 12)	192	(± 14)	196	(± 14)
HR_one_ (bpm)	162	(± 16)	160	(± 17)	161	(± 17)
HR_five_ (bpm)	105	(± 14)	105	(± 15)	105	(± 15)
HRR_one_ (bpm)	39	(± 13)	37	(± 12)	38	(± 13)
HRR_five_ (bpm)	95	(± 11)	92	(± 11)	94	(± 11)

*Values are presented Mean (± SD). BF, Body fat; BMI, body mass index; VO_2max,_ Maximal oxygen update; HR_*rest*,_ resting heart rate; HR_*max*,_ maximal HR; HR_*one*,_ HR at 1 min; HR_*five*,_ HR at 5 min; HRR_*one*,_ HR recovery at 1 min; HRR_*five*,_ HR recovery at 5 min.*

### Replication of Previously Established Methods of Assessment of RR_rec_

Results from replication of the methods established in Goldberger et al. (2006) are provided in Table 2 and visual representation of means from the segmented-analysis throughout the first 5 min of recovery are provided in Figure 2.

**TABLE 2:** *P*-values from ANCOVAs assessing differences for sex and race after controlling for body fat (BF), maximal oxygen uptake (VO_2p__eak_), and resting heart rate (HR_rest_) for each of the segmenting-analyses of post-exercise HRV.

	**15 s**			**30 s**			**60 s**		
	**SDNN**	**rMSSD**	**RMS**	**SDNN**	**rMSSD**	**RMS**	**SDNN**	**rMSSD**	**RMS**
Sex	0.43	0.36	0.45	0.45	0.41	0.44	0.74	0.76	0.59
Race	0.44	0.40	0.40	0.73	0.43	0.49	0.79	0.46	0.69
BF (%)	0.42	0.26	0.31	0.38	0.21	0.43	0.20	0.10	0.36
VO_2p__eak_ (ml/kg/min)	<0.001	<0.001	<0.001	<0.001	<0.001	<0.001	<0.001	<0.001	<0.001
HR_rest_ (bpm)	<0.001	<0.001	<0.001	<0.001	<0.001	<0.001	<0.001	<0.001	<0.001
Time	0.08	<0.001	0.001	0.07	<0.001	<0.001	0.75	<0.001	<0.001
Sex:BF	0.35	0.36	0.46	0.36	0.41	0.40	0.43	0.73	0.44
Sex:Time	0.11	0.46	0.42	0.52	0.24	0.20	0.72	0.14	0.65
BF:Time	0.24	0.03	0.45	0.24	0.02	0.20	0.98	0.04	0.65
Race:Time	0.21	0.74	0.54	0.66	0.86	0.22	0.14	0.38	0.26
VO_2p__eak_:Time	0.23	0.07	0.21	0.07	0.01	0.01	0.78	<0.001	0.005
HR_rest_:Time	0.20	0.05	0.45	0.65	0.01	0.32	0.24	<0.001	0.39
Sex:BF:Time	0.28	0.58	0.55	0.52	0.41	0.40	0.53	0.17	0.84

Replication of these previously established methods in our young adults provided similar results to those of the healthy middle-aged adults reported in Goldberger et al. (2006). Extending on the comparisons previously made between healthy individuals and those with coronary artery disease (Goldberger et al., 2006), we examined the relationships between sex and race after controlling for BF, VO_2p__eak_, and HR_rest_. While neither SDNN, rMSSD, or RMS were different between sex or race with any of the three segment-lengths, we did observe significant relationships between VO_2p__eak_ and HR_rest_ with each of these analyses. More interestingly were the significant interactions between VO_2p__eak_ and HR_rest_ with the changes in SDNN, rMSSD, and RMS across time and the lack of consistency in these findings across different segment-lengths (Table 2).

### Differencing and Polynomial Detrending of RR_rec_

*Pearson* correlations among demographic and the detrending (differencing- and residual-approaches) measures for the entire 5 min time series immediately post-exercise are presented in Table 3. The correlations between demographic characteristics and SDNN, rMSSD, and RMS of differenced and residual methods produced correlations ranging from −0.67 to 0.65. Similarly, the correlations between traditional indices of HRR with SDNN, rMSSD, and RMS ranged from −0.83 to 0.75.

**TABLE 3:** Correlations among demographic and the *differencing*- and *residual*- detrending measures.

	**BF**	**VO_2p__eak_**	**HR_rest_**	**HR_max_**	**HR_one_**	**HR_five_**	**HRR_one_**	**HRR_five_**	**SDNN_diff_**	**rMSSD_diff_**	**ApEn_diff_**	**SampEn_diff_**	**SDNN_res_**	**rMSSD_res_**	**ApEn_res_**
VO_2p__eak_	–0.78^‡‡^														
HR_rest_	0.27^†^	−0.29^†^													
HR_max_	−0.31^†^	0.47^‡‡^	0.12												
HR_one_	−0.21*	0.36^‡^	0.25*	0.78^‡‡^											
HR_five_	–0.05	0.20*	0.37^‡^	0.72^‡‡^	0.84^‡‡^										
HRR_one_	–0.03	–0.04	−0.27^†^	–0.1	–0.69^‡‡^	–0.50^‡‡^									
HRR_five_	−0.32^†^	0.29^†^	–0.38^‡‡^	0.21*	−0.21*	–0.52^‡‡^	0.59^‡‡^								
SDNN_diff_	0.12	−0.29^†^	−0.26^†^	–0.62^‡‡^	–0.76^‡‡^	–0.83^‡‡^	0.49^‡‡^	0.41^‡‡^							
rMSSD_diff_	0.09	−0.23*	−0.28^†^	–0.58^‡‡^	–0.72^‡‡^	–0.79^‡‡^	0.47^‡‡^	0.41^‡‡^	0.99^‡‡^						
ApEn_diff_	0.17	–0.06	0.39^‡‡^	0.43^‡‡^	0.60^‡‡^	0.70^‡‡^	–0.46^‡‡^	–0.47^‡‡^	–0.51^‡‡^	–0.51^‡‡^					
SampEn_diff_	−0.22*	0.31^†^	–0.06	0.26^†^	0.24*	0.26*	–0.08	–0.04	–0.55^‡‡^	–0.55^‡‡^	−0.29^†^				
SDNN_res_	0.20*	−0.36^‡^	−0.26^†^	–0.67^‡‡^	–0.81^‡‡^	–0.87^‡‡^	0.53^‡‡^	0.42^‡‡^	0.91^‡‡^	0.86^‡‡^	–0.50^‡‡^	–0.45^‡‡^			
rMSSD_res_	0.12	−0.29^†^	−0.26^†^	–0.62^‡‡^	–0.76^‡‡^	–0.83^‡‡^	0.49^‡‡^	0.41^‡‡^	1.00^‡‡^	0.99^‡‡^	–0.51^‡‡^	–0.55^‡‡^	0.91^‡‡^		
ApEn_res_	–0.15	0.28^†^	0.32^†^	0.65^‡‡^	0.68^‡‡^	0.75^‡‡^	−0.34^‡^	−0.26^†^	–0.70^‡‡^	–0.68^‡‡^	0.55^‡‡^	0.41^‡‡^	–0.75^‡‡^	–0.70^‡‡^	
SampEn_res_	−0.28^†^	0.40^‡‡^	0.04	0.37^‡^	0.32^†^	0.41^‡‡^	–0.09	–0.13	–0.38^‡‡^	−0.33^‡^	0.08	0.45^‡‡^	–0.56^‡‡^	–0.38^‡‡^	0.63^‡‡^

VO_2max_, Maximal oxygen update; HR_rest_, resting HR; HR_max_, maximal HR; HR_one,_ HR at 1 min; HR_five_, HR at 5 min; HRR_one_, HR recovery at 1 min; HRR_five_, HR recovery at 5 min; SDNN, standard deviation of the normal RR-interval; rMSSD, root mean square of successive differences; ApEn, approximate entropy; SampEn, sample entropy. Differencing method (_diff_); residual method (_res_).^‡‡^p < 0.0001; ^‡^p < 0.001;^†^p < 0.01; *p < 0.05.

Means from the newly proposed detrending methods are provided in Table 4 and results from the ANCOVAs are provided in Table 5. Similar to our findings with the segmenting-method, we did not observe significant group differences based on sex or race but we did observe significant associations between VO_2p__eak_ and HR_rest_ with SDNN, rMSSD, ApEn, and SampEn for all but SampEn_diff_.

**TABLE 4:** Means (± SD) of the *differencing-* and *residual- methods.*

	**Differencing**	**Residual**
SDNN	6.27 (± 4.70)	13.38 (± 7.47)
rMSSD	9.41 (± 6.95)	6.27 (± 4.70)
ApEn	1.09 (± 0.30)	1.10 (± 0.09)
SampEn	2.27 (± 0.39)	1.57 (± 0.28)

*Standard deviation of the normal RR-interval (SDNN); root mean square of successive differences (rMSSD); approximate entropy (ApEn); sample entropy (SampEn).*

**TABLE 5:** *P*-values from ANCOVAs assessing differences for sex and race after controlling for body fat (BF), maximal oxygen uptake (VO_2p__eak_), and resting heart rate (HR_rest_) for each of the *differencing-* and *residual-* methods.

	**Differencing**				**Residual**			
	**SDNN**	**rMSSD**	**ApEn**	**SampEn**	**SDNN**	**rMSSD**	**ApEn**	**SampEn**
Sex	0.82	0.75	0.19	0.03	0.41	0.82	0.71	0.17
Race	0.48	0.33	0.26	0.95	0.66	0.48	0.42	0.80
BF	0.20	0.28	0.43	0.09	0.72	0.20	0.38	0.34
VO_2p__eak_	<0.001	0.003	0.08	0.09	<0.001	<0.001	0.007	0.02
HR_rest_	<0.001	<0.001	0.001	0.44	<0.001	<0.001	<0.001	0.05
Sex:BF	0.69	0.63	0.29	0.12	0.31	0.68	0.73	0.52

Results from ARS models are provided in Table 6. The *residual-*method provided a significantly (*p* = 0.04) lower mean standard error (MSE) (0.064 ± 0.042) compared to the *differencing-*method (0.100 ± 0.033).

**TABLE 6:** Parameters from the multivariate adaptive regression spline (ARS) models for each of the *differencing-* and *residual-methods.*

	**Differencing**		**Residual**	
	**Parameter**	**Estimate**	**Parameter**	**Estimate**
SDNN	H(206-HR_max_)	0.033	H(BF-29.9)	0.011
	H(HRR_one_-26)	0.012	H(HR_one_-169)	–0.014
	H(98-HRR_five_)	–0.026	H(106-HR_five_)	0.036
	H(HRR_five_-98)	0.035	H(HR_five_-106)	–0.012
*R*^2^		*0*.*79*		*0*.*86*
*MSE*		*0*.*084*		*0*.*067*
rMSSD	H(21.5-BMI)	0.120	H(206-HR_max_)	0.033
	H(52-HR_rest_)	0.037	H(HRR_one_-26)	0.012
	H(206-HR_max_)	0.031	H(98-HRR_five_)	–0.026
	H(98-HRR_five_)	–0.020	H(HRR_five_-98)	0.035
	H(HRR_five_-0.042)	0.042		
*R*^2^		*0*.*77*		*0*.*79*
*MSE*		*0*.*127*		*0*.*084*
ApEn	H(37.2-BF)	–0.009	H(HR_max_-192)	0.027
	H(50-HR_rest_)	–0.029	H(HR_max_-195)	–0.023
	H(HR_rest_-50)	–0.042	H(HRR_five_-88)	–0.004
	H(HR_rest_-57)	0.051		
	H(HR_max_-192)	0.135		
	H(HR_max_-195)	–0.133		
	H(95-HRR_five_)	0.023		
*R*^2^		*0*.*73*		*0*.*61*
*MSE*		*0*.*061*		*0*.*005*
SampEn	H(18.5-BF)	0.044	H(52-HR_rest_)	0.033
	H(90-HRR_five_)	–0.037	H(32-HR_one_)	0.022
	H(HRR_five_-90)	–0.026	H(HRR_one_-35)	0.054
			H(HRR_one_-44)	–0.077
			H(HRR_five_-95)	–0.016
*R*^2^		*0*.*39*		*0*.*47*
*MSE*		*0*.*128*		*0*.*100*

*SDNN, Standard deviation of the normal RR-interval; rMSSD, root mean square of successive differences; ApEn, approximate entropy; SampEn, sample entropy; BF, body fat; HR, heart rate; HR_rest_, resting HR; HR_max_, maximal HR; HR_one_, HR at 1 min; HR_five_, HR at 5 min; HRR_one_, HR recovery at 1 min; HRR_five_, HR recovery at 5 min; MSE, mean square error.*

*Estimates for parameters where the variable precedes the hinge-point [e.g., H(HR_rest_-60)] represent the slope of the line after the hinge-point whereas estimates for parameters where the hinge-point precedes the variable [e.g., H(60-HR_rest_)] represent the slope of the line before the hinge-point.*

## Discussion

The major findings from these analyses were (1) reproducibility of previously established methods (Goldberger et al., 2006) for immediate post-exercise HRV in a young-adult population, (2) a unique dichotomy in the simple correlations between traditional measures of HRR and the complexity measures from the *differencing-* and *residual-*methods of RR_rec_, and (3) reliable models that demonstrate the complex nature of these indices. Additionally, these results provide preliminary insight into what physiologic variables *may* contribute to differences in these complexity indices when calculated on the detrended time series from the 5 min immediately post-exercise compared to traditional indices of HRR.

The replication of the *segmenting-method*, as described in Goldberger et al. (2006), (used to assess acute changes in RR_rec_) in our young adult population mirrored the patterns of change in SDNN, rMSSD, and RMS previously reported by Goldberger et al. (2006), however, the true raw values assessed for SDNN, rMSSD and RMS across the 5 min segment appeared to be different. This discrepancy may be associated with the difference in age between the two samples as our sample included adolescents and young-adults compared to Goldberger et al. (2006) which included adults and older-adults. The individuals included in our sample have completed puberty and linear growth by this age, and these data fall within the normal range of values for young healthy adults. However, these individuals are still developing psychologically and physiologically, which may contribute to these differences.

The ANCOVAs testing for effects with the segmenting-method, as well as the new residual-method (from the entire 5 min period immediately following the cessation of exercise) proposed in this paper, produced similar results: no effects for sex or race were observed for any of the tests, while both VO_2p__eak_ and HR_rest_ were significantly associated with SDNN, rMSSD, and RMS from both methods.

The correlations among various demographic variables and the variability and complexity indices from the *differencing-* and *residual-methods* in Table 3 show a unique relation between VO_2p__eak_ and HR_rest_ with HRR_one_, SampEn_diff_, and SampEn_res_. While the variability measures (SDNN_diff_, SDNN_res_, rMSSD_diff_, and rMSSD_res_) were each significantly correlated with traditional metrics of HRR (VO_2p__eak_, HR_rest_, HR_max_, HR_one_, HR_five_, HRR_one_, and HRR_five_), SampEn_diff_ and SampEn_res_ had either no correlation with, or much-lower correlations with these indices. These findings suggest that indices of variability calculated on the entire 5 min segment using either differencing or residual methods, share large amounts of variance with traditional indices of HRR—in other words, the information provided by assessing either SDNN_diff/res_ or rMSSD_diff/res_ does not provide innovative or novel information beyond the traditional indices of HRR investigated in this, and many other, studies. Considering the physiological mechanisms associated with sympathetic withdrawal and the role of vagal input following the cessation of exercise to reduce HR, these observations are in-line with what is expected.

However, the complexity metrics obtained surrounding the increase in RR_rec_ (i.e., SampEn_diff_, and SampEn_res_) immediately following the cessation of exercise are differentially correlated with demographic measures and various traditional indices of HRR. This suggests that the complexity indices may provide distinct and unique information about the changes in HR immediately following exercise. While the ANCOVAs further supported these observations, the best support for this idea came from the ARS models (Table 6). Contrary to the ANCOVA analyses, the ARS models are exploratory and do not test specific hypotheses. These models progressively add predictors (and any combination of predictors), to the model and test for various non-linearities among them. The optimal model is determined by comparing changes in model fit. The optimal model was calculated on a *training-dataset* (a randomly selected 50% of the entire dataset) and then tested on the *test-dataset*.

Both SampEn_diff_ and SampEn_res_ were significantly associated with VO_2p__eak_ and neither was significantly correlated with HR_rest_, HRR_one_, or HRR_five_, but these variables (or some combination of these variables) were repeatedly selected in the ARS models as significant predictors of the complexity metrics (ApEn_diff_, ApEn_res_, SampEn_diff_, and SampEn_res_). Similarly, HR_rest_, HRR_one_, and HRR_five_ were commonly identified by ARS as significant terms in other models, suggesting that the *variability* surrounding the trend in RR_rec_ immediately following the cessation of exercise does not provide information about changes in cardiac autonomic regulation that is not already observed in traditional indices of HRR. However, the repeated selection of HR_rest_, HRR_one_, and HRR_five_ by ARS models to predict SampEn_diff_, or SampEn_res_ despite the lack of significant correlations between any of these variables is an interesting finding that highlights the novelty of these detrending methods. These findings suggest that the *complexity* surrounding the trend in RR_rec_ provides information about cardiac autonomic regulation not detected through traditional HRR indices or the variability surrounding the trend in RR_rec_.

Although the *segmenting-method* can be used to separate out the *fast-phase* and the *slow-phase* of the cardiac autonomic response to the cessation of exercise, we suggest that assessing the entire 5 min together may provide a more holistic and comprehensive assessment of the cardiac dynamics associated with RR_rec_. Furthermore, calculating the residuals from a third-order polynomial fit to the first 5 min of RR_rec_ is less computationally intense and likely to be more robust against acute changes in RR_rec_ compared to the segment approach previously described (Goldberger et al., 2006). When considering data length, it’s important to remember that although time is a constant, there could potentially be drastic differences in the number of RR-intervals that occur within each segment being analyzed. Furthermore, the influence of irregular and/or erroneous beats would have a larger impact on the variability (McNames et al., 2003; McNames and Aboy, 2006; Thuraisingham, 2006) and complexity (Rhea et al., 2011) score calculated across a shorter segment when compared to a longer, 5 min, sampling period. Thus, we recommend the *residual-method* (as outlined in our methods) as a method of assessing the cardiac dynamics associated with recovery from exercise. In line with the findings of Berry et al. (2020) this method also produced more accurate and reliable models compared to the *differencing-method—*observed through a significantly higher MSE in the ARS models calculated on the *differenced* data compared to the *residual* data.

Although the time course for parasympathetic reactivation post-exercise has been investigated previously, the exact timing of these changes is likely to be highly impacted by an individual’s health and training status and thus, to vary from person to person. As such, the interindividual variability in post-exercise parasympathetic reactivation may provide a nuanced assessment of cardiac autonomic regulation and subtle changes in this exercise-induced responsiveness may reflect the early stages of disease—an important target for future studies. While the development of an algorithm capable of reliably parsing-out these points in the recovery phase is feasible, automating this analysis would be computationally intensive—*more-so* than the method (SampEn_res_) outlined here, which we have shown can be predicted by non-linear relations among demographic information and traditional measures of HRR.

Several other methodological approaches have been suggested in the scientific literature to address HRV assessment immediately post-exercise, but a variety of concerns and confounds (Task-Force, 1996; Rhea et al., 2011; Peçanha et al., 2017), such as segment length, significantly limit the utility of these metrics in the exercise literature. Importantly, while many of these metrics are mathematically rigorous and computationally more feasible than ever before, the crux of their utility in exercise physiology hinges on their ability to translate into meaningful physiologic contexts. Herein lies one of the major advantages to complexity assessments surrounding the RR_rec_, since it translates easily into a meaningful physiological construct. Highly adaptive systems show increasing entropy (greater complexity) and are known to be associated with healthy responses (Shaffer and Ginsberg, 2017) and increased adaptability of the system (West and Goldberger, 1987; Manor et al., 2010).

In summary, we assessed the variability and complexity surrounding the non-stationary trend in RR_rec_ through two methods including differencing and residual approaches. Results of SDNN, rMSSD, and SampEn calculations from the two detrending methods were similar, however, the multivariate ARS models produced from the residual approach had a lower MSE when compared to the differencing approach. This suggests that information about the individual recovery response immediately following the cessation of maximal exercise may be better represented through *(third-order)* polynomial regression detrending methods compared to differencing approaches. The complexity surrounding the trends in RR_rec_ immediately following the cessation of exercise provides unique information and novel context related to cardiac autonomic regulation and the dynamics of RR_rec_ not observed through traditional measures of HRR. Whereas the variability surrounding RR_rec_ does not provide additional information beyond traditional HRR metrics already utilized in the literature. Further research is needed to establish the utility of this approach in other settings (i.e., field) and in other populations (i.e., older adults, clinical populations and athletes) as well as investigating other psychophysiological factors that may contribute to the dynamic regulation of cardiac control following the cessation of exercise. In addition, the relations of other commonly utilized metrics (e.g., spectral analyses) within the HRV literature should be applied to these methods.

## Data Availability Statement

The raw data supporting the conclusions of this article will be made available by the authors, without undue reservation.

## Ethics Statement

The studies involving human participants were reviewed and approved by the Institutional Review Board at University of North Carolina at Greensboro. The patients/participants provided their written informed consent to participate in this study.

## Author Contributions

NB: data collection, data analysis, statistical analysis, and writing. EB: writing and editing. LS, SK, and SC: project design and editing. LW: project design, data collection, and editing. All authors contributed to the article and approved the submitted version.

## Conflict of Interest

The authors declare that the research was conducted in the absence of any commercial or financial relationships that could be construed as a potential conflict of interest.

## Abbreviations

HR: Heart Rate
HRR: Heart Rate Recovery
HRV: Heart Rate Variability
RR_rec_: RR Intervals During Recovery
SDNN: Standard Deviation of RR-intervals
rMSSD: Root Mean Square of Successive RR Differences
ApEn: Approximate Entropy
SampEn: Sample Entropy
RMS: Root Mean Square Index
VO_2peak_: Peak Oxygen Uptake
diff: Difference Calculation
res: Residual Calculation
MSE: Mean Square Error
ARS: Adaptive Regression Splines.

## References

[B1] Arai Y. Saul J. P. Albrecht P. Hartley L. H. Lilly L. S. Cohen R. J. (1989). Modulation of cardiac autonomic activity during and immediately after exercise. *Am. J. Physiol. Heart Circ. Physiol.* 256 H132–H141. 10.1152/ajpheart.1989.256.1.H132 2643348

[B2] Bernardi L. Salvucci F. Suardi R. Soldá P. L. Calciati A. Perlini S. (1990). Evidence for an intrinsic mechanism regulating heart rate variability in the transplanted and the intact heart during submaximal dynamic exercise? *Cardiovasc. Res.* 24 969–981. 10.1093/cvr/24.12.969 2097063

[B3] Berry N. T. Wideman L. Rhea C. K. (2020). Variability and complexity of non-stationary functions: methods for post-exercise HRV. *Nonlinear Dyn. Psychol. Life Sci.* 24 367–387. 32960753

[B4] Breuer H. W. Skyschally A. Schulz R. Martin C. Wehr M. Heusch G. (1993). Heart rate variability and circulating catecholamine concentrations during steady state exercise in healthy volunteers. *Heart* 70 144–149. 10.1136/hrt.70.2.144 8038025 PMC1025275

[B32] Fox J. Weisberg S. (2019). *An R Companion to Applied Regression*, 3rd Edn. Thousand Oaks, CA: SAGE Publications Available online at: http://socserv.socsci.mcmaster.ca/jfox/Books/Companion

[B5] Fox J., Weisberg S. *Multivariate Linear Models in R. 31.*.

[B6] Friedman J. H. (1991). Multivariate adaptive regression splines. *Ann. Stat.* 19 1–67. 10.1177/096228029500400303 8548103

[B7] Ganio M. S. Klau J. F. Casa D. J. Armstrong L. E. Maresh C. M. (2009). Effect of caffeine on sport-specific endurance performance: a systematic review. *J. Strength Cond. Res.* 23 315–324. 10.1519/jsc.0b013e31818b979a 19077738

[B8] Goldberger J. J. Le F. K. Lahiri M. Kannankeril P. J. Ng J. Kadish A. H. (2006). Assessment of parasympathetic reactivation after exercise. *Am. J. Physiol. Heart Circ. Physiol.* 290 H2446–H2452. 16415073 10.1152/ajpheart.01118.2005

[B9] Goldberger Ary L. Amaral Luis A. N. Glass L. Hausdorff J. M. Ivanov P. C.h. Mark R. G. (2000). PhysioBank, PhysioToolkit, and PhysioNet. *Circulation* 101 e215–e220. 10851218 10.1161/01.cir.101.23.e215

[B10] Karapetian G. K. Engels H. J. Gretebeck R. J. (2008). Use of heart rate variability to estimate LT and VT. *Int. J. Sports Med.* 29 652–657. 10.1055/s-2007-989423 18213538

[B11] Lachman S. Terbraak M. S. Limpens J. Jorstad H. Lucas C. Scholte op, et al. (2018). The prognostic value of heart rate recovery in patients with coronary artery disease: a systematic review and meta-analysis. *Am. Heart J.* 199 163–169. 10.1016/j.ahj.2018.02.008 29754656

[B12] Manor B. Costa M. D. Hu K. Newton E. Starobinets O. Kang H. G. (2010). Physiological complexity and system adaptability: evidence from postural control dynamics of older adults. *J. Appl. Physiol.* 109 1786–1791. 10.1152/japplphysiol.00390.2010 20947715 PMC3006415

[B13] McNames J. Aboy M. (2006). Reliability and accuracy of heart rate variability metrics versus ECG segment duration. *Med. Biol. Eng. Comput.* 44 747–756. 10.1007/s11517-006-0097-2 16960742

[B14] McNames J. Thong T. Goldstein B. (2003). “Reliability and accuracy of heart rate variability metrics versus ECG segment duration,” in *Proceedings of the 25th Annual International Conference of the IEEE Engineering in Medicine and Biology Society* (IEEE Cat No03CH37439), Vol. 1 (Cancun), 212–215.

[B15] Michael S. Graham K. S. Davis G. M. O. (2017). Cardiac autonomic responses during exercise and post-exercise recovery using heart rate variability and systolic time intervals—a review. *Front. Physiol.* 8:301 10.3389/fphys.2017.00301/full PMC5447093 28611675

[B16] Milborrow S. (2017). *earth: Multivariate Adaptive Regression Splines. R package version 460.*

[B17] Nakamura Y. Yamamoto Y. Muraoka I. (1993). Autonomic control of heart rate during physical exercise and fractal dimension of heart rate variability. *J. Appl. Physiol.* 74 875–881. 10.1152/jappl.1993.74.2.875 8458809

[B18] Ng J. Sundaram S. Kadish A. H. Goldberger J. J. (2009). Autonomic effects on the spectral analysis of heart rate variability after exercise. *Am. J. Physiol. Heart Circ. Physiol.* 297 H1421–H1428. 19648255 10.1152/ajpheart.00217.2009 PMC2770769

[B19] Oida E. Moritani T. Yamori Y. (1997). Tone-entropy analysis on cardiac recovery after dynamic exercise. *J. Appl. Physiol.* 82 1794–1801. 10.1152/jappl.1997.82.6.1794 9173943

[B20] Peçanha T. Bartels R. Brito L. C. Paula-Ribeiro M. Oliveira R. S. Goldberger J. J. (2017). Methods of assessment of the post-exercise cardiac autonomic recovery: a methodological review. *Int. J. Cardiol.* 227 795–802. 10.1016/j.ijcard.2016.10.057 27836300

[B21] Perini R. Orizio C. Baselli G. Cerutti S. Veicsteinas A. (1990). The influence of exercise intensity on the power spectrum of heart rate variability. *Eur. J. Appl. Physiol.* 61 143–148. 10.1007/bf00236709 2289492

[B22] Perini R. Veicsteinas A. (2003). Heart rate variability and autonomic activity at rest and during exercise in various physiological conditions. *Eur. J. Appl. Physiol.* 90 317–325. 10.1007/s00421-003-0953-9 13680241

[B23] Qiu S. Cai X. Sun Z. Li L. Zuegel M. Steinacker J. M. (2017). Heart rate recovery and risk of cardiovascular events and all−cause mortality: a meta−analysis of prospective Cohort studies. *J. Am. Heart Assoc.* 6:e005505. 10.1161/JAHA.117.005505 PMC5524096 28487388

[B24] Rhea C. K. Silver T. A. Hong S. L. Ryu J. H. Studenka B. E. Hughes C. M. L. (2011). Noise and complexity in human postural control: interpreting the different estimations of entropy. *PLoS One.* 6:e17696. 10.1371/journal.pone.0017696 21437281 PMC3060087

[B25] Rodríguez-Liñares L. Vila X. Méndez A. J. Lado M. J. (2008). “R-HRV: an R-based software package for heart rate variability analysis of ECG recordings,” in *Proceedings of the 3rd Iberian Conference in System and Information Technologies*, Ourense, 565–574.

[B26] Shaffer F. Ginsberg J. P. (2017). An overview of heart rate variability metrics and norms. *Front. Public Health* 5:258 10.3389/fpubh.2017.00258/full PMC5624990 29034226

[B27] Task-Force (1996). Task force of the European society of cardiology and the North American society of pacing and electrophysiology. *Circulation* 93 1043–1065. 8598068

[B28] Thuraisingham R. A. (2006). Preprocessing RR interval time series for heart rate variability analysis and estimates of standard deviation of RR intervals. *Comput. Methods Programs Biomed.* 83 78–82. 10.1016/j.cmpb.2006.05.002 16806571

[B29] West B. J. Goldberger A. L. (1987). Physiology in fractal dimensions. *Am. Sci.* 75 354–365.

[B30] Wideman L. Calkins S. D. Janssen J. A. Lovelady C. A. Dollar J. M. Keane S. P. (2016). Rationale, design and methods for the RIGHT track health study: pathways from childhood self-regulation to cardiovascular risk in adolescence. *BMC Public Health* 16:459. 10.1186/s12889-016-3133-7 27246836 PMC4888421

[B31] Yentes J. M. Hunt N. Schmid K. K. Kaipust J. P. McGrath D. Stergiou N. (2013). The appropriate use of approximate entropy and sample entropy with short data sets. *Ann. Biomed. Eng.* 41 349–365. 10.1007/s10439-012-0668-3 23064819 PMC6549512

